# Utilizing nanozymes for combating COVID-19: advancements in diagnostics, treatments, and preventative measures

**DOI:** 10.1186/s12951-023-01945-9

**Published:** 2023-06-21

**Authors:** Jia Wang, Qingpeng Xie, Haoyue Song, Xiaohang Chen, Xiaoxuan Zhang, Xiangyu Zhao, Yujia Hao, Yuan Zhang, Huifei Li, Na Li, Kelong Fan, Xing Wang

**Affiliations:** 1 Shanxi Medical University School and Hospital of Stomatology, Taiyuan, 030001 China; 2 Shanxi Province Key Laboratory of Oral Diseases Prevention and New Materials, Taiyuan, 030001 China; 3 CAS Engineering Laboratory for Nanozyme, Key Laboratory of Protein and Peptide Pharmaceutical, Institute of Biophysics, Chinese Academy of Sciences, Beijing, 100101 China

**Keywords:** Nanozymes, COVID-19, SARS-CoV-2, Nanomedicine

## Abstract

The emergence of human severe acute respiratory syndrome coronavirus 2 (SARS-CoV-2) poses significant challenges to global public health. Despite the extensive efforts of researchers worldwide, there remains considerable opportunities for improvement in timely diagnosis, specific treatment, and effective vaccines for SARS-CoV-2. This is due, in part, to the large number of asymptomatic carriers, rapid virus mutations, inconsistent confinement policies, untimely diagnosis and limited clear treatment plans. The emerging of nanozymes offers a promising approach for combating SARS-CoV-2 due to their stable physicochemical properties and high surface areas, which enable easier and multiple nano-bio interactions in vivo. Nanozymes inspire the development of sensitive and economic nanosensors for rapid detection, facilitate the development of specific medicines with minimal side effects for targeted therapy, trigger defensive mechanisms in the form of vaccines, and eliminate SARS-CoV-2 in the environment for prevention. In this review, we briefly present the limitations of existing countermeasures against coronavirus disease 2019 (COVID-19). We then reviewed the applications of nanozyme-based platforms in the fields of diagnostics, therapeutics and the prevention in COVID-19. Finally, we propose opportunities and challenges for the further development of nanozyme-based platforms for COVID-19. We expect that our review will provide valuable insights into the new emerging and re-emerging infectious pandemic from the perspective of nanozymes.

**Graphical Abstract:**

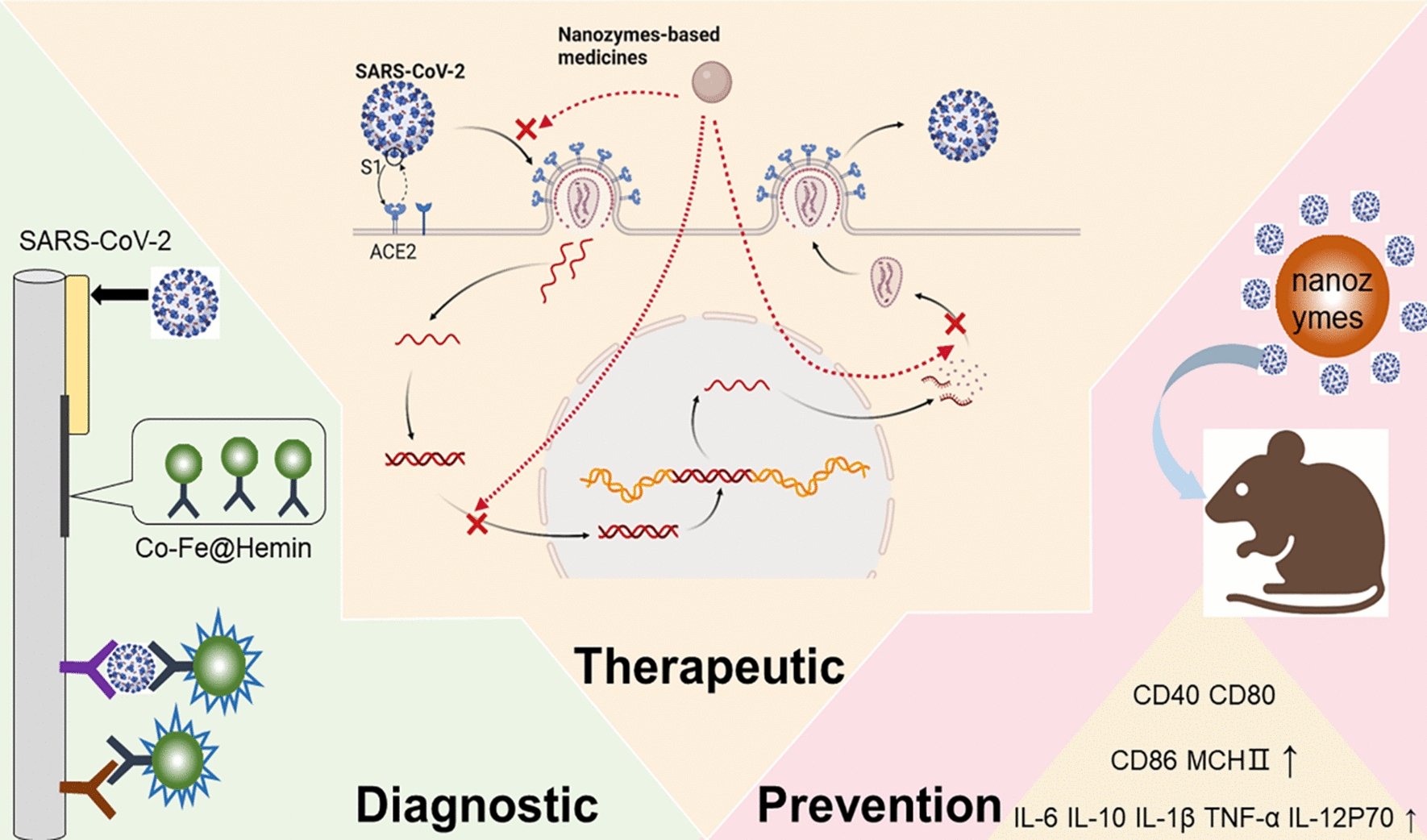

## Highlights

1. Nanozyme-based platforms have demonstrated improved sensitivity for SARS-CoV-2 antigen detection, reduced cost, and facilitated rapid diagnosis, making them a promising tool for COVID-19 diagnostics.
2. Nanozyme-based platforms offer potential benefits in targeted therapy for COVID-19 due to their ability to specifically counteract and eliminate SARS-CoV-2 with potentially fewer side effects.
3. Nanozyme-based platforms have been shown to act as immunostimulatory molecules, activating the defense response of the immune system against SARS-CoV-2, potentially providing a therapeutic avenue for COVID-19 treatment.

## Introduction

As of May 22, 2023, over 765 million individuals have been affected by severe acute respiratory syndrome coronavirus 2 (SARS-CoV-2), resulting in over six million confirmed deaths. SARS-CoV-2 is extremely infectious and can be transmitted between people within six feet [1]. At the present stage, most infections are mild or moderate and may have cough, fever, headache, nasal congestion and shortness of breath [2]. However, long-term SARS-CoV-2 infection will inevitably produce sequelae, chest radiographic abnormalities, impaired pulmonary diffusion capacity or depression symptoms [3]. Once an elderly patient who normally has underlying diseases is infected with SARS-CoV-2, the "cytokine storm" in the body after infection may lead to acute respiratory distress syndrome (ARDS), severe sepsis or septic shock, and even multiorgan dysfunction [4]. In response to the coronavirus disease 2019 (COVID-19) epidemic caused by SARS-CoV-2, there is still large amounts of works that need to be done. (1) Rapid diagnostic methods are still lacking [5]. Nucleic acid-based testing recommended by the World Health Organization (WHO) is time-consuming and complicated [6]. (2) Current treatments or medicines are not targeted []. Many of the approved drugs are aimed primarily at symptom relief [7]. (3) Effective and secure COVID-19 vaccines remain urgently needed [8]. At this stage, SARS-CoV-2 continues to mutate to obtain stronger infectivity [9]. In particular, Omicron variants can escape the protection of developed antibodies. In such a complex scenario, there is a greater urgency to develop rapid detection capability as well as to deploy targeted therapies and create more effective vaccines to prevent the progression of COVID-19 [10].

Enzymes, with significant catalytic activity, control the basic metabolic and life-sustaining activities of biological systems on the earth [11]. Most natural enzymes are spherical proteins or bioorganic molecules, which are easily affected by environmental factors such as temperature, pH or humidity [11]. Nanozymes are particular nanomaterials with inherent mimetic enzyme properties, combining the strengths of nanomaterials and natural enzymes [12]. Nanozymes exhibit higher stability, tunable catalytic activity, multienzyme activity, smart response and self-assembly capability [13]. To date, a great number of nanozymes have been developed to imitate the natural ones, such as peroxidase (POD), oxidase (OXD), catalase (CAT), and superoxide dismutase (SOD) [14]. In recent years, nanozymes have shown great application prospects in biomedicine, such as antimicrobial activity, biosensing, disease detection and cancer treatment [15].

**Fig. 1:**
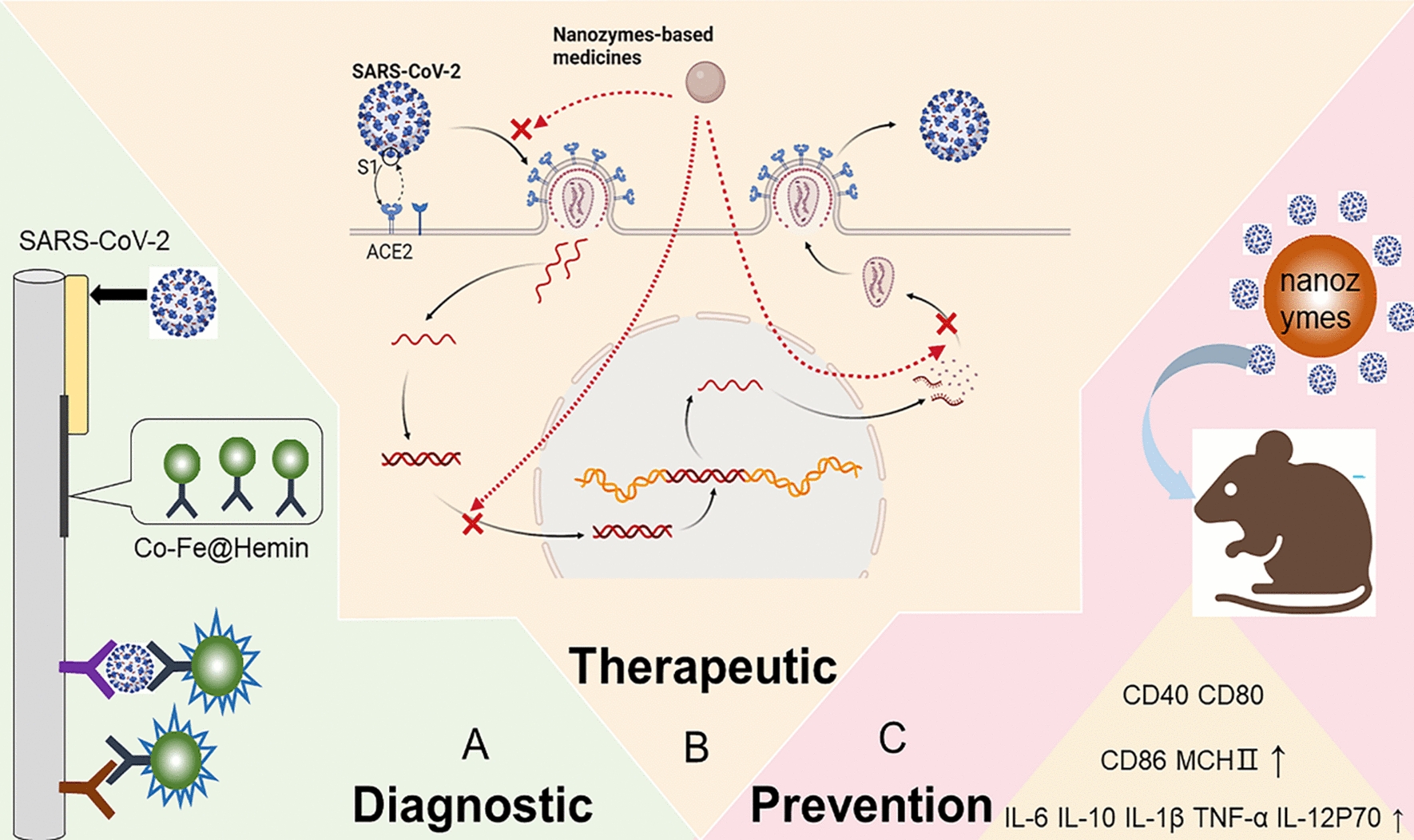
Summary of nanozyme-based platforms in combating COVID-19. **A** Nanozyme chemiluminescent paper assay for SARS-CoV-2 antigen in diagnosis. **B** Possible mechanism of nanozyme-based medicines preventing SARS-CoV-2 infection in therapeutic. **C** Nanozyme‐based mucosal vaccines are necessary to prevent COVID-19. Created with BioRender.com

In the past few decades, nanozymes have shown their great effectiveness in fighting against various viruses. Duan et al*.* utilized Fe_3_O_4_ magnetic nanoparticles (MNPs) as a nanozyme probe to detect Ebola virus [16]. The sensitivity of the nanozyme strip is 100 times higher than that of the standard strip method, and it is much faster and simpler. Qin et al*.* targeted the lipid envelope of influenza virus and destroyed the integrity of neighboring proteins by using iron oxide nanozymes (IONzymes), causing its inactivation [17]. Inspired by the wide antiviral applications of nanozymes, many scholars believe that nanozymes have great potential in treating SARS-CoV-2 infections [18]. (1) Nanozyme-based platforms can improve the sensitivity of antigen testing of SARS-CoV-2, reduce the cost and be conducive to rapid diagnosis. (2) Nanozyme-based platforms can assist in targeted COVID-19 treatment, specifically by resisting and clearing SARS-CoV-2 and producing fewer side effects. (3) Nanozyme-based platforms may act as immunostimulatory molecules to activate the defense responses of the immune response to SARS-CoV-2. However, few reviews have summarized the possible contribution of nanozymes in fighting against COVID-19 (Fig. 1).

In this review, we discussed the limitations of existing countermeasures in combating COVID-19. Moreover, we reviewed the applications of nanozyme-based platforms in the fields of diagnosis, treatment and prevention of COVID-19 and their safety considerations. Finally, we proposed opportunities and challenges for the further development of nanozyme-based platforms for COVID-19. We expect that our paper will put forward new opinions in facing the COVID-19 pandemic from the perspective of nanozymes (Table 1).

**Table 1:** Application of nanozymes in COVID-19

Nanozymes	Catalytic Activity	Application	References
Co–Fe@hemin	Peroxidase-like activity	Rapid and sensitive detection of SARS-CoV-2 antigen	[29]
Au@Pt	Peroxidase-like activity	Colorimetric detection of spike protein of SARS-CoV-2	[47]
FeS_2_	High peroxidase-like activity	Rapid and ultrasensitive nucleic acid detection of SARS-CoV-2	[123]
MnO_2_	Oxidase-like activity	Point-of-care testing of SARS-CoV-2	[124]
Au@Pt	Peroxidase-like activity	Quantitative detection of SARS-CoV-2 nucleocapsid protein	[125]
Pt@Au	Peroxidase-like activity	Quantitative detection of SARS-CoV-2	[36]
Pd-Ir	Peroxidase activity	Nucleocapsid protein from SARS-CoV-2	[126]
Ag-TiO_2_	Peroxidase-like activity	Inhibition of SARS-CoV-2 entry into cells	[127]
MCeC@MΦ	Superoxide dismutase and catalase-like activities	Treatment of cytokine storm caused by COVID-19	[128]
CuNPs	Photo-activity	Confers self-sterilizing ability and reusability to masks	[116]
TiO2	Photo-activity	Clears SARS-CoV-2 from the surface of an item	[129]

## Current diagnosis and nanozyme-based diagnosis of COVID-19

### Current testing for SARS-CoV-2

When a new infectious pathogen appears, quick and precise diagnosis is considered to be the primary requirement for quickly tracking and isolating cases [19]. Early diagnoses with good sensitivities and high accuracies are very important when COVID-19 is spreading throughout an area, but practical detection options for the identification of SARS-CoV-2 are somewhat limited. There are three main ways to detect SARS-CoV-2 infections: nucleic acid detection, antibody detection, and antigen detection [20].

#### Principles of current detection

Among these techniques, the reverse transcription-polymerase chain reaction (RT‒PCR) method in nucleic acid detection is the gold standard for early diagnosis of COVID-19 [21]. Viral RNA is extracted from a sample of a person’s nose or throat after treatment with chemical solutions [22]. Reverse transcriptase and primers will reverse-transcribe the viral RNA to single-stranded complementary DNA (cDNA) [23]. Then, DNA polymerase extends the second strand, generating double-stranded DNA. DNA is amplified continuously in the RT‒PCR machine. Organic dyes are connected with the DNA strand and release fluorescence. When the fluorescence exceeds a certain level, the patient is considered to be contracted with SARS-CoV-2. It was reported that when diagnosing SARS-CoV-2, the sensitivity of PCR can reach 0.14 copy/μL [24], and the specificity can reach 96–100%. Antibody testing is another way to diagnose COVID-19 [25]. Antibodies are derived from the immune system as it combats SARS-CoV-2, suggesting that the body is fighting pathogens [26]. The detection of antibodies can be achieved by serologic testing by measuring the IgG and IgM levels in blood or plasma, which are produced by neutralizing virus antigens [27]. IgM is the first antibody to appear as a first immune response, suggesting a recent infection, and can be used as an aid in the diagnosis of early infection. IgG is produced later, as it lasts longer and is mostly used as an indicator of previous or subsequent infections [28]. Seroconversion kinetics vary considerably between antibody detection kits. In summary, the serum sample and sample diluent are added to the test strip, left flat at room temperature for about 10 min and the results are then evaluated according to the colour of the test and control lines [28]. The antigen test is based on the recognition of SARS-CoV-2 proteins and is used to identify the active replication virus in the early stages of infection. Antigen testing methods utilize techniques similar to serological methods, using enzyme-linked immunosorbent assays (ELISA) or chemiluminescent immunoassays [5]. As point of care devices, antigen testing methods can be conducted by minimally trained personnel in various primary and even community environments, with test results provided within 5–30 minutes and obtainable in a single clinical contact.

#### Limits of the current diagnosis scheme

Although nucleic acid detection is greatly encouraged by the WHO, it requires biosafety laboratories, professional instruments and skilled experts [18]. Moreover, it takes at least 1–2 hours to complete RNA extraction, reverse transcription, gene amplification and data analysis [29]. Hence, nucleic acid detection is not suitable for on-site and real-time screening [30]. At the same time, nucleic acid detection is very difficult for many poor neighborhoods or health caring systems, particularly in developing nations, due to the high cost.

Regarding antibody detection, the results may be false-positive due to cross-reactivity when the antibody is bound to an antigen that is distinct from the targeted antigen [31]. The reason for the cross reaction is the similarity between the molecules. Antibodies are only produced after at least 10 days of virus infection [32], so this general late response may lead to false-negatives [33]. Negative results of antibody tests may not confirm that the patient is not infected [34]. At the same time, serological testing as an invasive method is not convenient and not suitable for rapid detection at all. It appears that serologic tests are better suited for extensive screening of patients with infection but are not suitable for early diagnosis of COVID-19 [35].

Compared with nucleic acid testing or antibody testing, antigen detection can detect the virus itself, which is convenient for large-scale population screening [36]. However, most antigen tests can provide only qualitative results, not quantitative results [37]. ELISA based on antigen determination is very time-consuming and has complex washing and incubation steps [38].

It is necessary to control the incidence of COVID-19 by quick diagnosis of emerging patients [39]. There is still an urgency to have a simpler, faster, more cost-effective and more reliable approach for SARS-CoV-2 antigen identification [40].

### Nanozyme-based detection of SARS-CoV-2

Nanozymes with high surface volume ratios enable them to have large reservoirs and abundant anchoring sites to load and deliver agents, so they can diagnose various virus infections quickly, sensitively and in real time [41]. It may be a good idea to apply nanozymes to the rapid diagnosis of COVID-19 in antigen diagnosis.

#### Nanozymes for chemiluminescence paper test

**Fig. 2:**
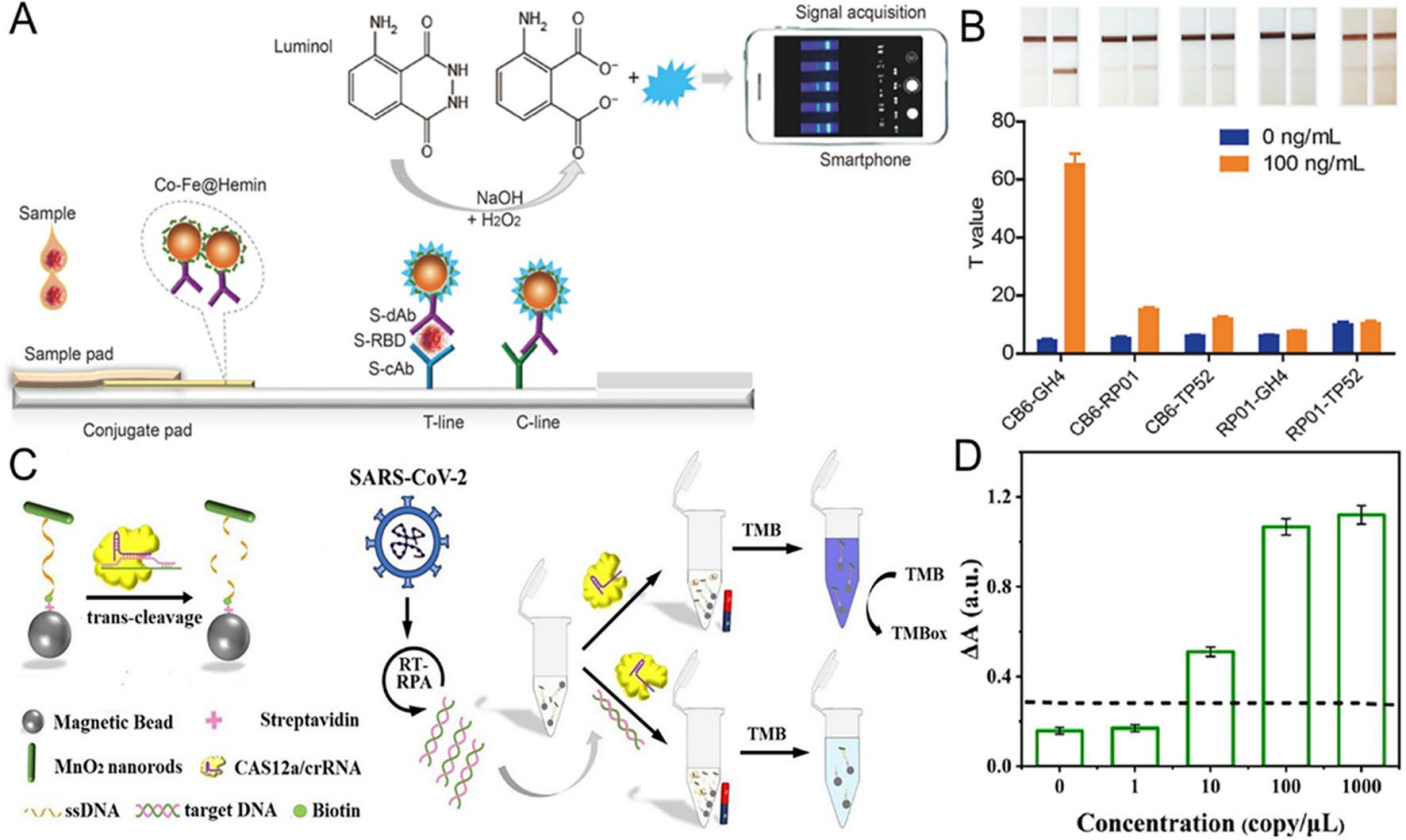
Nanozyme-based detection of SARS-CoV-2. **A** Schematic illustration of the Co–Fe@hemin nanozyme chemiluminescent paper test for SARS-CoV-2. **B** Screening of paired antibodies using a Co–Fe@hemin nanozyme colorimetric strip. A positive signature was rated by 100 ng/mL of S-RBD protein. **C** A protocol for detecting SARS-CoV-2 using the CRISPR‒Cas12a system based on the ssDNA-MnO_2_-MB reporter. **D** Detecting differing levels of SARS-CoV-2 E pseudovirus based on the RPA-conjugated MnO_2_ nanozyme-mediated CRISPR‒Cas12a system. (Copyright 2020. Biosens Bioelectron, Copyright 2022, ACS Appl Mater Interfaces)

Traditional antigen detection is mainly based on colloidal gold immune assays or fluorometric immunochromatographic assays [42]. Liu et al*.* investigated a novel chemiluminescence paper using nanozymes for rapid and highly responsive determination of SARS-CoV-2 spike antigen [29] (Fig. 2A, B). Compared with traditional colloidal gold or fluorescent test paper and nucleic acid tests, this nanozyme chemiluminescence paper has the following advantages: (1) The key point of this test paper is a powerful Co-Fe@heme peroxidase nanoprobe, which promotes chemiluminescence and amplifies the immune reaction signal. Chemiluminescence signal amplification will improve the accuracy of the paper-based assay and reduce the incidence of false-negatives in earlier screening for SARS-CoV-2 disease. (2) The time taken for testing can be accomplished in less than 16 min, which is much faster than the nucleic acid test. (3) Importantly, they use Co–Fe@heme peroxidase nanozymes to replace natural horseradish peroxidase (HRP), which is the central material in conventional chemiluminescent immunodiagnostics [43]. Natural enzymes such as HRP are high-cost, unstable and complex to produce [44]. By comparison, Co–Fe@heme nanozymes are considerably more vigorous in hot and alkaline environments. Therefore, nanozyme chemiluminescent testing strips can be stably kept at environmental temperatures, which is conducive to their transportation and field application. (4) Nanozymes are relatively inexpensive and can be synthesized from easily available materials, while naturally occurring HRP needs complicated extraction and purification. As a result, the general cost of nanozyme-based chemiluminescent test paper is comparatively lower, which can greatly reduce the financial burden on national health care. This study is susceptible to errors from both reconstituted antigens and pseudoviruses, so it is important to further validate the accuracy of pharyngeal swab or nasal swab samples from infected persons. Parallel comparisons with other commercial kits for large-scale application are needed.

#### Nanozymes for colorimetric detection

Colorimetric assays have potential applications in detecting SARS-CoV-2. Bartolomeo et al. utilized gold nanoparticles (NPs) to cause a change in the extinction spectra of the relevant solutions within just a few minutes to obtain a rapid and credible determination of SARS-CoV-2 [45]. In addition, studies have shown that the addition of different metals to metal NPs to build polymetallic nanostructures contributes to an improvement in enzyme activities, so developing polymetallic nanozymes has become an efficient tactic [46]. Zhao et al*.* utilized the brilliant POD catalytic action of polyclonal antibody-linked Au@Pt NPs to enable efficient and sensitive colorimetric analysis of the spike-in (S1) protein of SARS-CoV-2 [47]. They prepared homogeneous and monodispersed gold nanoparticles as their seeds and then added platinum atoms into the gold nanoparticles, which led to considerable modification of the enzymatic catalytic pathways from the production of reactive oxygen species (ROS) to the process of rapid electron transfer (ET). With the benefits of being fast, simple to handle and highly flexible, colorimetric assays based on Au@Pt nanoparticles can assist in the quick diagnosis of COVID-19. Their peroxidase catalysis is markedly strengthened by the multiporous nanostructures and electronic-rich Pt envelope of the Au@Pt NPs. The results of this experiment will contribute to the understanding of the catalytic mechanisms of nanozymes and the development of colorimetric biological sensors for actual diagnostics on the basis of metal NPs.

Clustered Regularly Interspaced Short Palindromic Repeats (CRISPR) and CRISPR-associated (Cas) based testing systems have been used to detect nucleic acids [48–52], proteins [53–55], and small molecules [56–58]. Fluorescent assays are often used in conjunction with CRISPR‒Cas. Wu et al*.* built a nanozyme-mediated colorimetric detection method for SARS-CoV-2 using a CRISPR‒Cas12a-based system [59] (Fig. 2C, D). Manganese dioxide nanostrips were used as a reporting tool on the basis of oxidative enzyme-like activities. Active Cas12a mediates the reverse fragmentation of single-stranded DNA linkers between MnO_2_ nanorods and label magnetic beads in the presence of SARS-CoV-2, which reduces the oxygenation of tetramethylbenzidine, triggering color variation. The application of MnO_2_ nanozymes in place of naturally occurring enzymes allows the usage of H_2_O_2_ to be avoided, making it cost-effective and simple to operate. In addition, chromatographic analyses based on nanozymes and CRISPR‒Cas12a systems allow the measurement to be easily manipulated and are suitable for high volume microtiter-based analyses. The MnO_2_ nanozyme-guided CRISPR‒Cas12a system could be used as an outstanding tool for testing SARS-CoV-2.

#### Nanozyme-linked immunochromatographic sensor for quantitative detection

Liang et al*.* developed a nanozyme-linked immunochromatographic sensor (NLICS) for the quantification of SARS-CoV-2 nucleocapsid protein antigens in human blood samples [60]. Au@Pt NPs, the nucleocapsid protein antigens in serum samples, the primary specific monoclonal antibodies (mAb1) and the secondary mAb2 form a sandwich construction (Au@PtNPs-mAb1-nucleocapsid protein-mAb2). Finally, the base material solution undergoes a calorific reaction mediated by the intercalated structure. The light filtered through the reaction matrix was measured by a light meter, and the outcome was transferred simultaneously to a microphone via a Bluetooth connection for data processing and analyses. This will be important for the prompt identification of suspected cases of SARS-CoV-2 infection, particularly at the early phases of virus exposure. Compared with other antigen detection methods, this is a quantitative detection method, and the diagnosis of infected people will become more accurate. It provides a fresh idea for the highly sensitive and quantitative diagnosis of COVID-19.

## Current therapeutics and nanozyme-based therapeutics against COVID-19

### Current medicines and treatment schemes against COVID-19

#### Medicines

The following categories of medicines are being investigated or are in development for the control of COVID-19: antiviral medicines (such as remdesivir and favepiravir), anti-inflammatories (dexamethasone and statins), antibodies (such as convalescent plasma and hyperimmune immunoglobulins), targeted immunomodulatory therapies (such as tocilizumab, sarilumab, anakina and ruxolitinib), anticoagulants (such as heparin), and antifibrotic medicines (such as tyrosine kinase inhibitors) [61]. Pfizer declared in November 2021 that Paxlovid taken within the first few days of symptom onset resulted in an 89% reduction in hospitalisation rates in high-risk patients [62].

#### Treatment schemes

Virus inhibition is the most effective measure in the earlier stage of the infection. Hydroxychloroquine is hypothesized to suppress viral entrance and endocytosis in vitro and may produce favorable effects of immunomodulation in vivo [63]. Lopinavir, an antiretroviral protease inhibitor, has shown some antiviral activities against SARS-CoV-2 [64]. Other antiviral medicines that may be used in COVID-19 are also being explored, including famotidine, favipiravir, arbidol and camostat (TMPRSS2 inhibitor) [65]. In the later stage, alternative therapeutic strategies are necessary; for example, immunomodulators may help to protect against progression of the disorder, and anticoagulants may be helpful to avoid comorbidities. Glucocorticoids can alleviate inflammation-mediated lung injury, thereby reducing the risk of progressing to respiratory failure and death. Dexamethasone has a greater survival benefit for COVID-19 patients who are recruited after the first week of their illness or received respiratory support [66]. It has been reported that therapy with methylprednisolone is also connected with decreased mortality risk. Taking monoclonal antibodies that target key inflammation mediator interferons such as beta-1b is conducive to alleviating symptoms, shortening hospital stay and preventing organ damage with mild to modest COVID-19 [67]. More than 75% of inpatients with COVID-19 require supplemental oxygen therapy. Alternative approaches that are being explored include subcutaneous low original weight heparin, nursing home plasma-derived super immunoglobulin and monoclonal antibodies against SARS-CoV-2 [68, 69].

#### Limits of current therapeutics

In the context of the quick spread of SARS-CoV-2 and the growing demand for a rapid response, a globally recognized specific treatment is still lacking. The main goal of current medicines is to control excessive inflammatory and immune reactions in serious conditions [70]. Most of these medicines are administered systemically, resulting in multiple medicine interactions. At the same time, these medicines are accompanied by many adverse reactions [71]. For instance, adverse reactions are higher in patients treated with hydroxychloroquine, and the most common is diarrhea [72]. Lopinavir can also induce hepatic injury [73]. Remdesivir has a broad spectrum of antiviral activities, including against coronaviruses and filoviruses. However, the actual drug delivery concentration that reaches the lungs, and more precisely, the host infected cells, is insignificant. With regard to Paxlovid, it must be taken immediately after the onset of symptoms. More importantly, its availability to low-income settings is very poor, making for huge differences in treatment opportunities [62, 74]. Therefore, with the rapid growth of the COVID-19 incidence rate, we urgently need to find more accurate treatment methods or medicines. More targeted drugs can not only reduce unnecessary drug consumption but also reduce side effects.

### Nanozyme-based treatment against COVID-19

As with most nanomaterials, nanozymes are also used as treatment options for diseases, such as serving as drug carriers or passively targeting diverse antiviral treatments [75]. Various forms of nanozymes possess considerable potential for antiviral applications [76]. A novel cold-adapted nanozyme based on a manganese-based nanosized metal-organic framework is developed to inactivate influenza virus H1N1 even at −20 ^°^C. Multiwall and single-wall carbon nanotubes, carbon dots and carbon nanodiamonds both have the potential to directly suppress virus reproduction [77]. Due to the benefits of both natural enzymes and nanomaterials, nanozymes have become a good choice for the treatment of COVID-19.

**Fig. 3:**
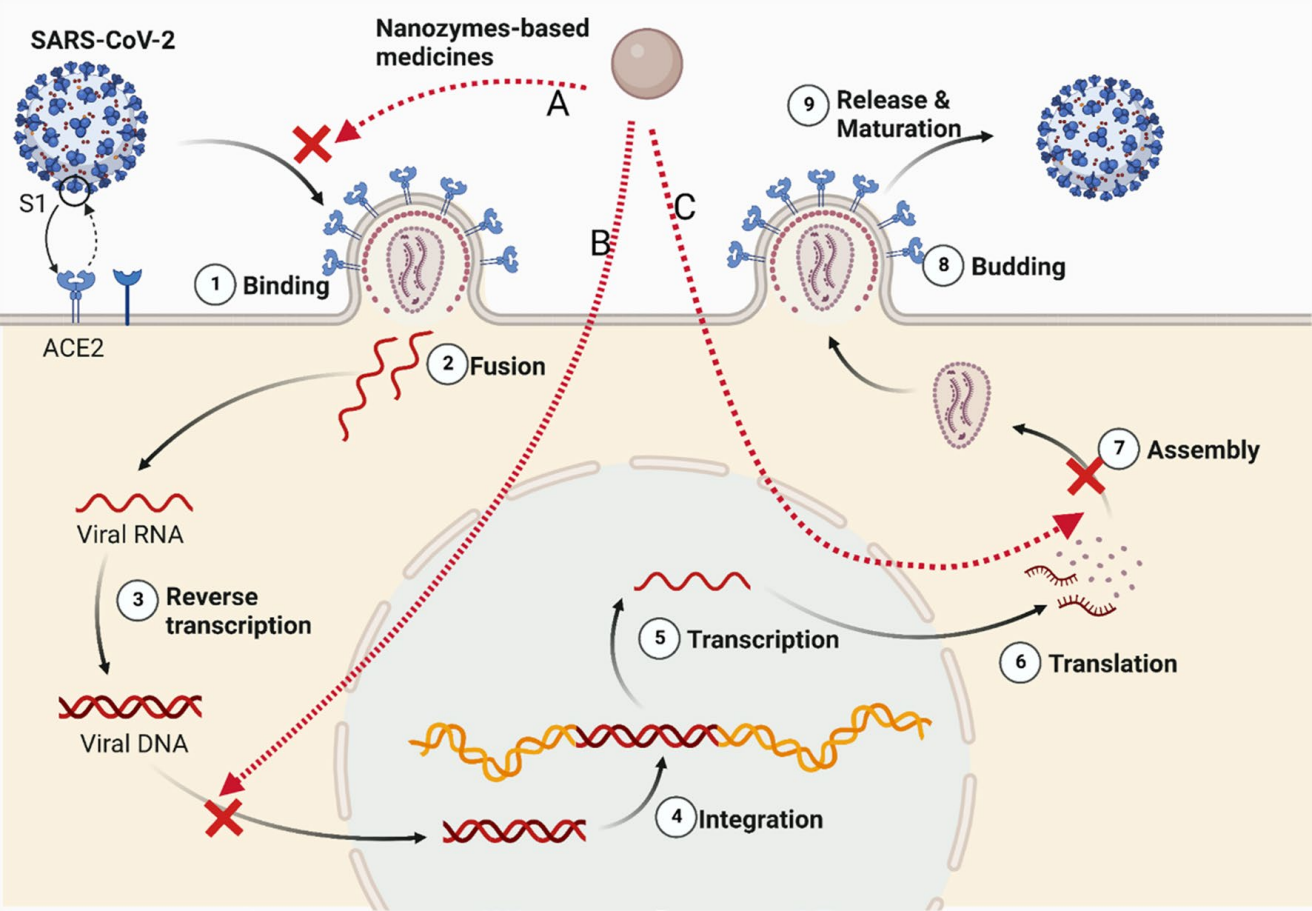
Possible mechanism of nanozyme-based medicines preventing SARS-CoV-2 infection. **A** Inhibiting the endocytosis of SARS-CoV-2 by nanozymes. **B** Blocking SARS-CoV-2 replication by nanozymes. **C** Blocking SARS-CoV-2 assembly and proliferation by nanozymes. Created with BioRender.com

The structural proteins of SARS-CoV-2 are composed of nucleocapsid protein (N), membrane protein (M), envelope protein (E), and spike protein (S). [78]. When SARS-CoV-2 comes into contact with the host cell, the S glycoprotein binds to the membrane receptor angiotensin-converting enzyme 2 (ACE2) and enters the host cell through endocytic action [79]. The S protein is cleaved into S1 and S2 subunits during their biosynthesis in the infected cells. The S1 fraction is involved in the recognition and adhesion of the cell membrane, and the S2 fraction is used for membrane fusion and entry into host cells [80]. Therefore, the inhibitory effect of nanozymes on endocytosis will become a strategic goal to inhibit SARS-CoV-2 infection. After the fusion of the virus envelope and endoplasmic membrane, the virus genome is liberated to the cytoplasm and transformed into RNA-dependent RNA polymerase (RdRp) for gene replication. The inhibition of replication by nanozymes following virus entry into host cells is also considered to be an effective therapeutic target (Fig. 3).

Due to reasons related to existing at the nanoscale, most nanozymes may have high affinity for virus attachment. Strong nano-bio interactions can recognize virus structures in situ. Nanozymes can interact with viral structures and directly inactivate or destroy them without harming host cells, which will minimize their side effects and degradation [81, 82]. Compared to traditional drugs, nanozyme agents typically have better biological distribution and increase accumulation in the target position, thus showing higher treatment effectiveness and fewer secondary effects [83].

#### Ag-TiO_2_ single-atom nanozymes for inhibiting SARS-CoV-2 entry

**Fig. 4:**
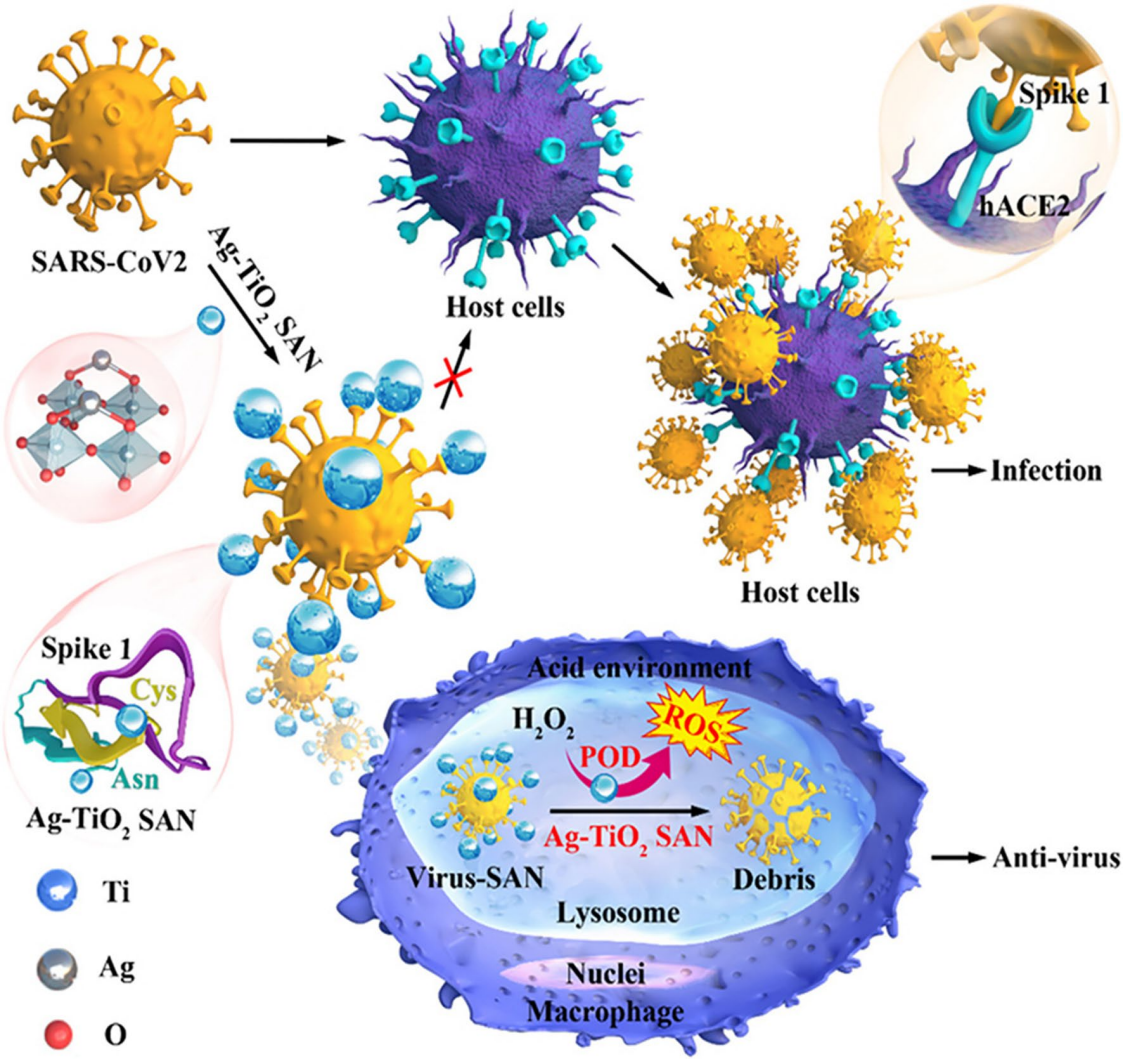
Schematic diagram of Ag-TiO_2_ SAN against SARS-CoV-2. The SAN Ag atom is tightly bound to both cysteine and asparagine, inhibiting the interaction between the S1 RBD and its receptor ACE2. (Copyright 2021, Nano Today)

Wang et al. successfully designed Ag-TiO_2_ single-atom nanozymes (SANs) with significant inhibition of SARS-CoV-2 entry activities (Fig. 4). The SAN Ag atom is tightly bound to both cysteine and asparagine, which are the two most enriched amino acids on the receptor binding domain (RBD) of the spike 1 protein of SARS-CoV-2, thereby inhibiting the interaction between the S1 RBD and its receptor ACE2 [84]. Then, the SAN/virus compound will be engulfed by macrophages and aggregated with lysosomes. Ag-TiO_2_ SAN initiates ROS production via POD-like activity to efficiently kill viruses. It can efficiently promote virus phagocytosis by macrophages in vivo and exhibit enhanced uptake with up to 99.65% for SARS-CoV-2 pseudovirus in vitro. Ag-TiO_2_ SAN can specifically resist and clear SARS-CoV-2 through its strong adsorptive property and POD-like activity, making up for the shortcomings of many existing broad-spectrum medicines. Ag-TiO_2_ SAN enables gram-level synthesis of a new SAN to combat and scavenge SARS-CoV-2, but it is used only in pseudoviruses of viral elimination models. Further verification of accuracy for real clinical samples is also needed.

#### ***V***_2_O_5_ nanozymes for inhibiting virus replication

Viral infection and replication are closely associated with oxidative stress. Singh et al*.* used vanadium pentoxide (*V*_2_O_5_) nanozymes to imitate natural glutathione peroxidase activity in function, catalyzing ROS neutralization in virus-infected cells to mitigate ROS and uniformly block viral reactivation and replication without adverse effects on cell physiology [85]. Meanwhile, increased levels of reactive oxygen or nitrogen species content are a distinctive characteristic of inflamed tissue. Nanozymes ROS scavenging ability can help the infected body relieve inflammation. The system is mainly applied to HIV‐infected individuals, and its use in SARS-CoV-2‐infected individuals is just around the corner. Researchers successfully revealed the potential usefulness of *V*^+^_2_O_5_ nanosheets against SARS-CoV-2 and suggested nanozymes as future platforms to develop interventions against COVID-19.

#### Nanozymes for the cytokine storm in COVID-19

During infection, excessive immune responses may cause overwhelming immune system impairment in the human body [86–88]. High levels of inflammatory cytokines such as interleukin (IL)-1β, IL-6, and tumor necrosis factor (TNF) and chemokines (C–C motif chemokine ligand (CCL)-2, CCL-3, and CCL-5) did not give good results in patients with poor outcomes [89–91]. Cytokine storms are the major contributor to ARDS in patients during COVID-19 infection and are an essential factor leading to COVID-19 aggravation or even death [4]. The decoy nanozymes, MCeC@MΦ, consist of mesoporous silica nanoparticle cores loaded with CeO_2_ nanocatalyst and Ce6 photosensitizer and biomimetic shells of macrophage membrane. The intense expression of cell membrane receptors in MCeC@MΦ allows the binding of decoy nanozymes to endotoxins or proinflammatory cytokines, which neutralizes endotoxin, sequesters proinflammatory cytokines and provides protection against overactivation of host immune cells [92]. At the same time, its superoxide dismutase, catalase-like activity and hydroxyl radical antioxidant capacity can be used to catalyze and remove multiple ROS in the body. MCeC@MΦ can significantly reduce the systemic hyperinflammatory response and rapid salvage of organ damage within 1 day in multidrug-resistant *Escherichia coli* bacteremia mice. This might open the possibilities of treating cytokine storms and immune-mediated inflammatory diseases caused by COVID-19.

## Current prevention and nanozyme-based prevention of COVID-19

Epidemiological data show that SARS-CoV-2 is mainly transmitted through airway splashing during intimate face-to-face exposure, such as talking, coughing or sneezing [93]. Other propagation modes of COVID-19 include aerosol propagation and contact surface propagation [94]. Compared to SARS-CoV-1, SARS-CoV-2 is extremely well stabilized in aerosols and on interfaces, remaining virally infectious for several hours [95].

### Current prevention measures for COVID-19

#### Daily defensive measures

Numerical modeling research and empirical data suggest that increasing the strength of public health care intervention can reduce the transmission rate of COVID-19 around the world [96–98]. Public health interventions include wearing masks and  restricting crowd aggregation in public places, enforcing social distancing and implementing centralized isolation after infection [97]. If a positive infection is found, the community should immediately expand the monitoring scale, implement contact tracing and isolate the infection to block the transmission chain [99]. However, the public health measures adopted by different countries may vary because of the influence of demographic, political, regional and other influencing factors [100]. Some countries may face challenges in effectively managing the COVID-19 pandemic due to the lack of medical resources. Moreover, only daily protection is not enough to control the transmission rate. The most important part of daily protective measures is wearing masks. Personal protective equipment, especially mask, is a key component of the strategies to prevent SARS-CoV-2 [101]. However, discarded disposable masks may become a source of rapid transmission of the virus. Meanwhile, the disposal and decontamination of masks have brought great environmental impacts and economic burdens [102].

#### Herd immunity and vaccines

With the COVID-19 pandemic, many countries have put forward the concept of “herd immunity” [103]. Herd immunity is achieved when a certain threshold of immunity at the population level is reached [104]. When the threshold of immunity is high enough and this threshold is met (whether through natural infection or vaccination), it can protect most people in a specific geographical region within a given time period and can break the chain of transmission of specific contagious diseases, in theory [105]. Specifically, herd immunity can be realized when the effective reproduction number R drops below 1 without any intervention, which means that an infected person in a group generates less than one secondary case on average[106].

Effective vaccination is the best way to accelerate herd immunity and eliminate epidemics without causing more deaths [107]. Recently, different types of vaccines have been authorized for worldwide clinical use to combat the COVID-19 pandemic [108]. COVID-19 is transmitted through the respiratory tract, and the mucosal immune response plays a part in the primary line of protection in the battle against pathogens, so mucosal vaccines have attracted the attention of many scholars in recent years [109]. Mucosal vaccines trigger robust mucosal and systemic immunity reactions at the major sites of viral exposure [110]. However, mucosal vaccine preparations need to ensure the immune availability and immune stimulation ability of vaccine antigens on the mucosal surface. Nasal barriers often obstruct the transmission of antigens to submucosal antigen presenting cells (APCs)‐dendritic cells (DCs) and restrict the subsequently mature level of DCs to activate protective adaptive immunity [111, 112]. An efficient antigen delivery system should be developed to protect the antigen of mucosal vaccines from degradation and clearance.

### Nanozyme-based prevention measures of COVID-19

#### CS‐IONzyme for mucosal vaccines

Nanozymes provide an ideal antigen delivery platform for vaccines, acting as adjacent platforms and mimicking virus structures, and they allow multivalent antigen presentation and antigen stability after administration [113]. Nanozymes can serve as adjuvants to enhance the immune response and initiate the defense system against SARS-CoV-2 to increase vaccine efficacy [114]. Nasal-based vaccines are a promising mucosal vaccine strategy for virus protection. Qin et al*.* developed a chitosan (CS)-functionalized iron oxide nanozyme (IONzyme)-based influenza whole inactivated virus (WIV) nasal vaccine [115]. CS‐IONzyme first increased the adherence level of H1N1 WIV to nasal mucosa and then vigorously motivated nasal epithelial cells to excrete the chemokine CCL20 while recruiting more dendritic cells to the submucosal area to form transepithelial dendrites (TED) for H1N1 WIV ingestion. Finally, dendritic cells complete antigen presentation and initiate the specific immune response. The adhesion of antigen to nasal mucosa was increased by 30 times, and the IgA-mucosal adaptive immunity was increased 8.9-fold. The CS-IONzyme-based nasal vaccine provided 100% protection against influenza with enhanced POD-like activity. Host infection with SARS-CoV-2 also occurs first through mucosal infection, so mucosal vaccines based on nanozymes are a new idea for COVID-19 vaccines. The study offers a prospective antiviral option for the design of IONzyme-based nasal vaccines to combat SARS-CoV-2.

#### Cu-nanozymes for photoactive antiviral face masks

**Fig. 5:**
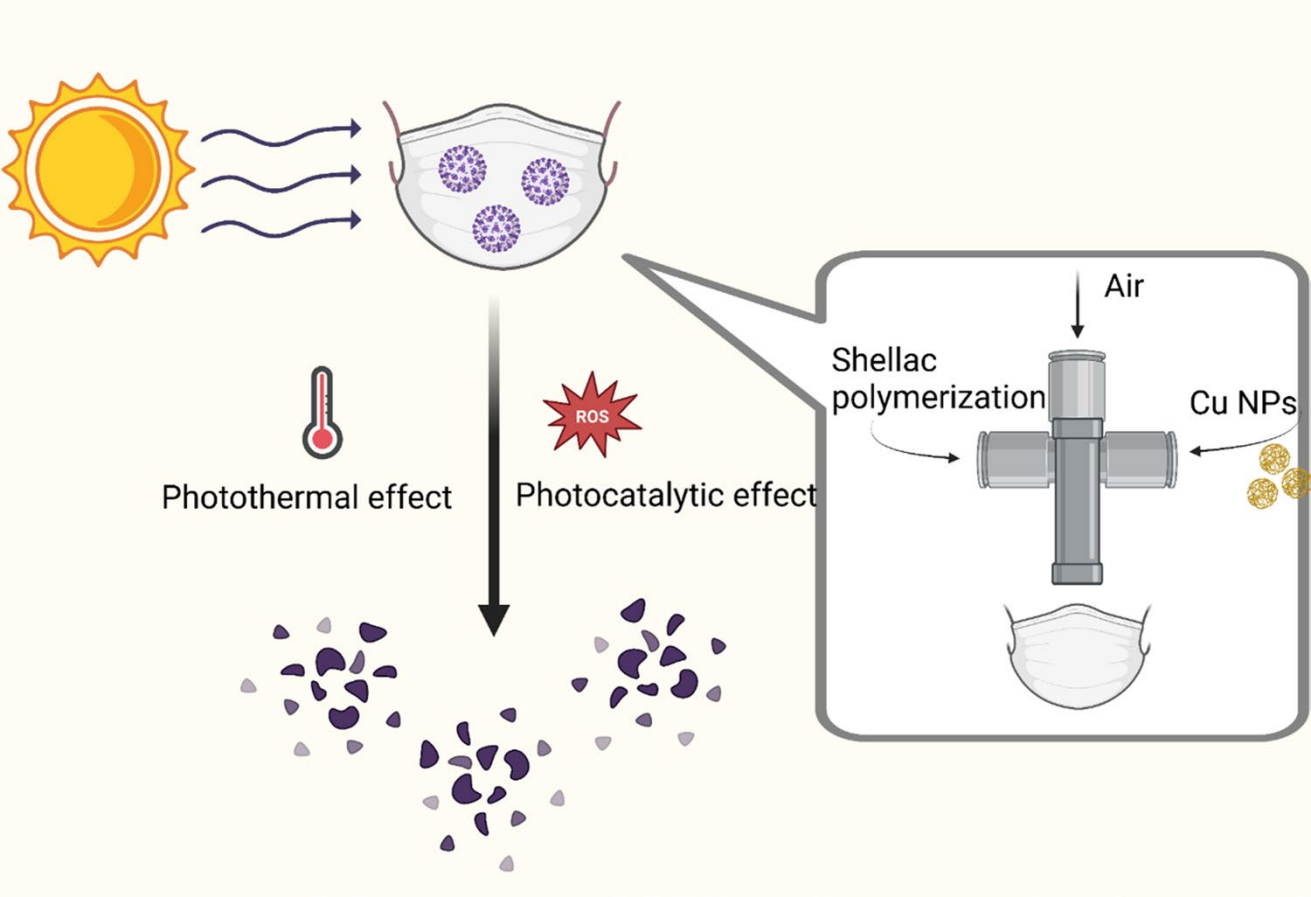
Cu-nanozymes for photoactive antiviral face masks. The manufacturing principle of the self-cleaning mask was as follows: the nebulizer was conceived to intermix CuNPs, shellac and pressurized air at the intersection. After solar irradiation, through photothermal effect and photocatalytic effect processes, the mask inactivates the virus and becomes self-cleaning. Created with BioRender.com

Kumar et al*.* sprayed a mixture of copper and shellac nanoparticles onto a nonwoven medical surgical mask, conferring self-sterilizing and reusability ability to the masks [116]. CuNPs enhanced the hydrophobicity of the mask and repelled water droplets, resulting in the mask exhibiting excellent photoactivity for antimicrobial action. Relying on the strong photocatalytic properties of copper nanozymes, these masks have fast and efficient bactericidal effects on pathogens. Under the sun, through photocatalytic and photothermal effects, masks can produce high levels of free radicals that disrupt the virus membrane, allowing the masks to be self-cleaning and reusable. These masks can prevent the spread of virus particles, provide important protection against the COVID-19 epidemic and contribute considerably to the abatement of financial expenses and environmental impacts (Fig. 5)**.**

#### Nanozyme-based surface coating against surface contact propagation

SARS-CoV-2 particles can remain on surfaces for a long time, which provide another route of transmission: surface contact [117]. Nanozymes can be sprayed onto various surfaces to improve the antiviral effectiveness of protective layers [118]. Some studies have explored the use of nanozymes as a means of decontaminating surfaces and reducing the spread of the virus. For example, photoactivated titanium dioxide successfully inactivated a wide spectrum of pathogens, including SARS-CoV-2, under a light irradiance level (0.4 mW/cm^2^ at a wavelength of 375 nm). Mechanistic research has shown that light-induced hydroxyl radicals target the viral genome, resulting in viral disintegration, instead of attacking the virion global structure and content [119]. Similarly, silver, gold, and copper nanozymes all have excellent abilities to damage viruses. Coronaviruses have been previously reported to remain viable on the object surface for up to 7 days, but SARS-CoV-2 viruses can only survive on copper surfaces for no more than 4 h [120]. Due to their increased contact with microbes by virtue of their small sizes, nanozymes are immensely effective for antiviral applications. The development of virus-resistant surfacing materials would be a powerful tool to protect against the transmission of COVID-19.

## Challenges of nanozymes in combating COVID-19

Despite nanozymes having numerous benefits for combating SARS-CoV-2, including high physical and chemical stability, excellent durability and simplicity, many challenges still remain.

One of the main challenges is the lack of knowledge about the underlying mechanisms of nanozymes drugs action with COVID-19. Additional studies are required to fully capture the biological and biochemical interactions between nanozymes and SARS-CoV-2 as well as the potential side effects and toxicity of these biocatalysts. Once nanozymes enter the human body, they can interact with biological molecules (mainly proteins), giving nanozymes new biological characteristics, which may be more significant than the original properties of nanozymes [121].

Another challenge is the lack of standardization in the design and production of nanozymes, which can lead to varying levels of efficacy and specificity. For instance, Au nanozymes are poisonous at 1.4 nm but not at 15 nm. It is crucial to closely explore the characteristic parameters of nanodrugs, particularly the relations among dimension, surface chemistry, dose and pharmacokinetics. Their physicochemical characteristics, such as dimension, geometry, electrical charge or surface chemistry, can be modified if necessary. Modulating the chemical composition and controlling the morphology to change the size, surface modification and assembly of nanozymes provide a reference for researchers in making nanozymes with maximum security for practical biomedical applications.

SARS-CoV-2 is constantly mutating [122], and nanozyme-based viral assays should still be validated using more complex clinical samples to obtain a more comprehensive and accurate diagnosis. Thus, simply using recombinant antigens or pseudoviruses is completely inadequate. The strategy of producing nanozyme diagnostic strips at scale is mandatory to increase their availability worldwide. This cost advantage can significantly reduce the financial burden on the nation's health care resources and, crucially, can benefit areas of poor socioeconomic status. Moreover, a parallel comparison of this strip assay with other commercially available SARS-CoV-2 kits is still required to make the nanozyme-mediated detection strategy commercially available.

To hasten the clinical adoption of nanozyme-based vaccines, a comprehensive assessment of their benefits and risks is essential. Safety is one of the most important concerns. Controlled dosage and surface modification can help to reduce toxicity and increase specificity for SARS-CoV-2, but their relevant influence on the following dynamics and catalytic activity of nanozymes must be evaluated. The immunogenicity and cellular transmigration of nanozymes must be researched. Regulation of reaction conditions at larger scales is challenging when producing nanozyme-based vaccines for SARS-CoV-2, so further expansion synthesis must be validated. Otherwise, issues such as batch variation will inevitably hinder the vaccines’ industrialization process.

## Conclusion

In the context of the SARS-CoV-2 global health outbreak, we have concluded the possible applications of nanozymes in fighting against SARS-CoV-2. (1) Regarding diagnosis, nanozyme-based detection provides a highly sensitive, rapid, inexpensive and even quantitative detection method compared to the current gold standard—the nucleic acid test. (2) In terms of treatment, they can specifically resist and clear SARS-CoV-2 by inhibiting its endocytosis or replication in the process of infecting host cells, which make up for the shortcomings of broad-spectrum medicines. Moreover, direct destruction of the viral structure can minimize its side effects and degradation. (3) Regarding prevention, nanozyme-based vaccines have better potential for appropriate exposure to the immune system and can protect vaccine antigens from degradation and removal. Nanozyme-based masks and surface coating will greatly reduce SARS-CoV-2 in the environment, reducing economic and environmental costs.

Although considerable progress has been made in recent years, there is still space for progress in the study of nanozymes in the fight against COVID-19. Here, we propose several key questions and directions of studies that can be researched in the future to increase the potential of clinical transformation of nanozymes. (1) For detection, to commercialize the nanozyme-mediated detection strategy, it is still necessary to evaluate nanozyme-based tests and other commercial kits. (2) For nanozyme-based medicines, we should evaluate their safety, pharmacokinetics, clinical toxicity, immunogenicity, and cellular fate. We should also consider the metabolism and subsequent catalytic activity of nanozymes in vivo. (3) For nanozyme-based vaccines, mass production and availability to the entire range of countries are the target directions. In conclusion, nanozyme-based tools are expected to play a frontline role in addressing COVID-19. Considering the continuous evolution of SARS-CoV-2, nanozymes must also "evolve" in morphology, composition and surface function to counteract.

## Data Availability

Not applicable.

## Abbreviations

SARS-CoV-2: Severe acute respiratory syndrome coronavirus 2
COVID-19: Coronavirus disease 2019
POD: Peroxidase
OXD: Oxidase
CAT: Catalase
SOD: Superoxide dismutase
ARDS: Acute respiratory distress syndrome
WHO: World health organization
MNPs: Magnetic nanoparticles
IONzymes: Iron oxide nanozymes
PCR: Polymerase chain reaction
RT‒PCR: Reverse transcription-polymerase chain reaction
cDNA: Complementary DNA
ELISA: Enzyme linked immunosorbent assay
HRP: Horseradish peroxidase
NPs: Nanoparticles
ROS: Reactive oxygen species
ET: Electron transfer
NLICS: Nanozyme-linked immunochromatographic sensor
CRISPR: Clustered regularly interspaced short palindromic repeats
Cas: CRISPR-associated
ACE2: Angiotensin converting enzyme 2
RdRp: RNA-dependent RNA polymerase
SANs: Single-atom nanozymes
RBD: Receptor binding domain
V_2_O_5_: Vanadium pentoxide
IL: Interleukin
TNF: Tumor necrosis factor
CCL: C–C motif chemokine ligand
APCs: Antigen presenting cells
DCs: Dendritic cells
CS: Chitosan
WIV: Whole inactivated virus
TED: Transepithelial dendrites
